# Healthy dietary patterns and metabolic dysfunction-associated fatty liver disease in less-developed ethnic minority regions: a large cross-sectional study

**DOI:** 10.1186/s12889-021-12486-x

**Published:** 2022-01-17

**Authors:** Xiaofen Xie, Bing Guo, Xiong Xiao, Jianzhong Yin, Ziyun Wang, Xiaoman Jiang, Jingzhong Li, Lu Long, Junmin Zhou, Ning Zhang, Yuan Zhang, Ting Chen, Baima Kangzhuo, Xing Zhao

**Affiliations:** 1 West China School of Public Health and West China Fourth Hospital, Sichuan University, No.16, People’s South Road, Chengdu, 610041 China; 2 School of Public Health, Kunming Medical University, Kunming, China; 3 Baoshan College of Traditional Chinese Medicine, Kunming, China; 4 School of Public Health, the key Laboratory of Environmental Pollution Monitoring and Disease Control, Ministry of Education, Guizhou Medical University, Guiyang, China; 5 Chengdu Center for Disease Control and Prevention, Chengdu, China; 6 Tibet Center for Disease Control and Prevention, Lhasa, China; 7 Chongqing Municipal Center for Disease Control and Prevention, No.8, Changjiang 2nd Road, Chongqin, 400042 China; 8 Tibet University, No.10, East Tibet University Road, Lhasa, 850000 China

**Keywords:** MAFLD, Metabolic, NAFLD, Dietary pattern, Obesity

## Abstract

**Background:**

Little is known about the associations between healthy dietary patterns and metabolic dysfunction-associated fatty liver disease (MAFLD) in less-developed ethnic minority regions (LEMRs), where the prevalence of MAFLD is increasing rapidly and dietary habits are quite different from those in developed countries. Moreover, a significant subset of MAFLD individuals in LEMRs are nonobese, but the efficacy of dietary patterns on MAFLD individuals with different obese statuses is also unclear. We aimed to test the associations of two wildly recommended *a priori* dietary patterns—Alternate Mediterranean diet (AMED) and Dietary Approaches to Stop Hypertension (DASH)—with the risk of MAFLD in the total population, and further in nonobese and obese individuals.

**Methods:**

We recruited 99,556 participants in the China Multi-Ethnic Cohort Study, an ongoing cohort study in less-developed southwest China. Using validated food frequency questionnaire, each participant was assigned an AMED score and a DASH score. MAFLD was ascertained as hepatic steatosis on ultrasound together with diabetes, overweight/obesity, or two other metabolic risk factors. We performed logistic regression with inverse probability of exposure weighting (IPEW) to examine associations between two dietary patterns and MAFLD, adjusting for potential confounders under the guidance of directed acyclic graphs. Further, analyses were stratified by body mass index.

**Results:**

We included 66,526 participants (age 49.5±11.0; 62.6% women), and the prevalence of MAFLD was 16.1%. Participants in the highest quintile of DASH score showed strong inverse associations with risks of MAFLD (*OR* = 0.85; 95% CI, 0.80-0.91; *P*_*trend*_ < 0.001) compared with participants in the lowest quintile. The association between DASH and nonobese MAFLD (*OR* = 0.69; 95% CI, 0.61-0.78; *P*_*trend*_ < 0.001) was stronger (*I*^2^ = 78.5 % ; *P*_*heterogeneity*_ = 0.001) than that with obese MAFLD (*OR* = 0.90; 95% CI, 0.83-0.98; *P*_*trend*_ = 0.002). There was a null association between AMED and MAFLD risk.

**Conclusions:**

In LEMRs, a DASH diet but not AMED was associated with MAFLD. The relationship appeared to be more pronounced in nonobese MAFLD individuals than in obese MAFLD individuals.

**Supplementary Information:**

The online version contains supplementary material available at 10.1186/s12889-021-12486-x.

## Background

Non-alcoholic fatty liver disease (NAFLD) is the most prominent chronic liver disease worldwide and affects approximately 25% of the global population [1]. Individuals with NAFLD may progress to fibrosis, cirrhosis, and hepatocellular carcinoma [2]. As it is largely associated with metabolic diseases, NAFLD has been recently renamed as metabolic dysfunction-associated fatty liver disease (MAFLD) with a consensus by an international panel of experts [3, 4]. Over the past decade, the prevalence of NAFLD has dramatically increased in less-developed countries [1, 5], especially in populations of low socioeconomic status (SES) and racial/ethnic minorities [6, 7], with the rate increasing more than twice as fast in some regions as in developed countries [8]. It has become a global public health concern to reduce the prevalence of MAFLD in less-developed regions.

Diet is one of the key modifiable lifestyle factors involved in the management of MAFLD, as with the previous term NAFLD [9]*.* Some studies have examined the effects of different dietary components or nutrients on the risk of NAFLD [10, 11]. However, individual dietary constituents are intercorrelated with each other to influence disease. By contrast, dietary patterns reflect the overall effect of diet and facilitate dietary recommendations [12]. Some healthy dietary patterns, such as the Mediterranean diet and the Dietary Approaches to Stop Hypertension (DASH) diet, have been recommended for the management of NAFLD [13, 14]. However, these dietary patterns were proposed based on dietary habits from developed countries, and whether they can be generalized to other populations, particularly racial/ethnic minority groups in less-developed regions, is still doubtful. To our knowledge, large population-based evidence on the efficacy of western dietary guidance in MAFLD management from less-developed ethnic minority regions (LEMRs) is scarce.

Furthermore, although MAFLD is strongly associated with obesity, it can also develop in individuals with a relatively normal body mass index (BMI), a condition that is often described as lean or nonobese MAFLD [4] and was initially described as nonobese NAFLD in Asian populations [15]. The prevalence of nonobese NAFLD in the US was reported to be 7% [16], while in some rural areas of Asian countries, it ranges from 4 to 40% [8]. Previous studies reported that nonobese NAFLD individuals may even be at higher risk for the development of severe liver disease than obese NAFLD [17] and associated with higher mortality [18]. However, there have been few data on the efficacy of dietary intervention in nonobese individuals with NAFLD or MAFLD, and it is still unclear whether clinicians should recommend dietary guidance to individuals with MAFLD in both nonobese and obese populations. Therefore, identifying the phenotype of MAFLD and related dietary interventions is of vital importance for the management of MAFLD.

Based on a large-scale population, using data from the China Multi-Ethnic Cohort (CMEC) Study with highly diverse socioeconomic status, ethnicity, habitual diet, cultural background, living environment, etc. [19], we aimed to (1) investigate the associations of two dietary patterns [alternate Mediterranean diet (AMED) score and Dietary Approaches to Stop Hypertension (DASH) score] with MAFLD risk in the total population and (2) examine the associations of the same dietary patterns in nonobese and obese individuals.

## Methods

### Study population

**Fig. 1:**
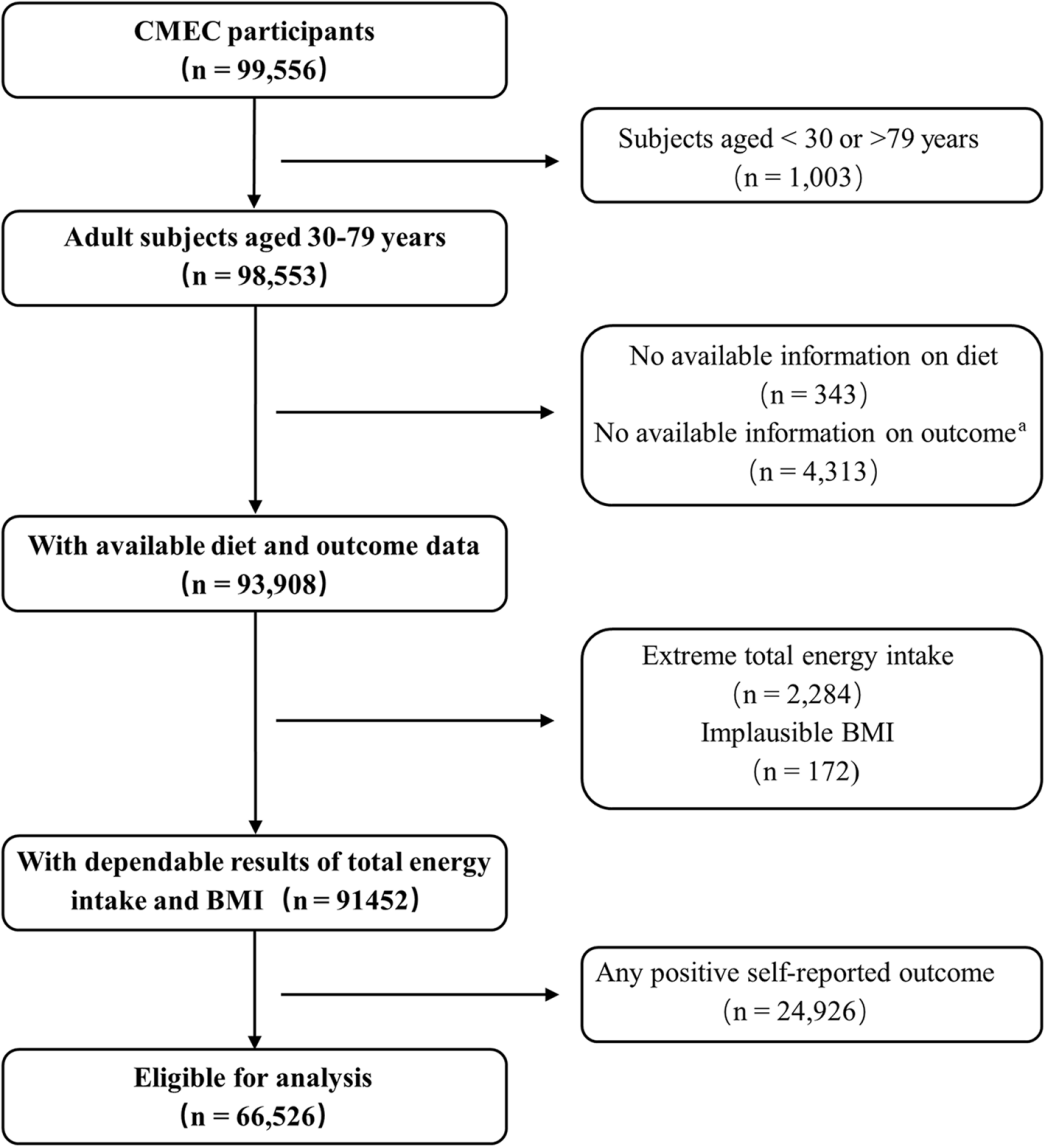
Flowchart of the study. CMEC, the China Multi-Ethnic Cohort Study; BMI, body mass index. ^a^ Outcome included physical measurement and blood tests

The CMEC study is a population-based prospective cohort study involving approximately 100,000 adults from six ethnic minority groups as well as the majority Han group in less-developed Southwest China. Details of the study design have been described previously [19]. In brief, 99,556 participants were recruited at a baseline survey between May 2018 and September 2019, given a full consideration of the diversity in SES, ethnic characteristics, and disease patterns. All participants completed a full laptop-based questionnaire with face-to-face interviews and underwent anthropometric measurement and blood tests by well-trained health workers. The flowchart of the study population is depicted in Figure 1. We excluded participants who were aged < 30 or >79 years at the baseline survey, had no available information on diet and outcome data, had extreme total energy intake (< 600 or > 3500 kcal/day for females and < 800 or > 4200 kcal/day for males), and had implausible BMI (< 14 or > 45 kg/m^2^). Furthermore, to eliminate potential reverse causality, 24,926 participants who self-reported physicians diagnosed with chronic hepatitis/cirrhosis, diabetes, hypertension, hyperlipidemia, coronary heart disease, stroke, or cancer at the baseline survey were excluded. Finally, a total of 66,526 eligible participants were included in the main analysis.

The study protocol was approved by the Sichuan University Medical Ethical Review Board and local ethics committee at each participating site. All the participants provided their written informed consent before data collection.

### Data on diet

Dietary information was collected at baseline using a quantitative food frequency questionnaire (FFQ) based on the Chinese Dietary Guidelines and the eating habits of Southwestern Chinese, covering 13 major food groups: rice, wheat products, coarse grain, tubers, red and processed meats, poultry, fish/sea food, eggs, dairy products, fresh vegetables, preserved vegetables, fresh fruits, and soybean products. Participants were asked how often on average during the preceding 12 months they had consumed each food group and how many grams per meal according to a standard serving size molds. For alcohol and sweeten beverage, the frequency, quantity, and types of consumption were collected; for oil and salt, the household consumption and how many persons eat at home at breakfast, lunch, dinner during the past month were asked. Finally, we calculated the daily consumption of each food group and alcohol as well as the average consumption of cooking oil and salt per person. Total energy intake was estimated based on the China food exchange lists and the 2018 China food composition tables (standard edition) [20, 21]. In addition, to assess the reproducibility, the FFQ was repeated during the repeated survey from August 2020 to November 2020 in nearly 10% of the baseline participants. Additionally, in a subsample of 1,163 participants, we conducted a three-day nonconsecutive 24-hour dietary recall (24HDR) to assess the validity of the FFQ. The Spearman rank correlation coefficients for reproducibility between the FFQ food groups ranged from 0.16 for fresh vegetables to 0.62 for alcohol and tea, and the de-attenuated Spearman rank correlation coefficients for validity ranged from 0.1 for soybean products to 0.66 for rice. More details are provided in our previous study [22].

### Scores of dietary patterns

Scores for the two dietary patterns (i.e., AMED and DASH) were computed for all participants. The AMED score was designed to assess adherence to a Mediterranean dietary pattern based on an adapted version of traditional Mediterranean diet for a non-Greek population [23–25]. We focused on 8t key food or nutrient group components: fruits, vegetables, legumes, whole grains, fish, ratio of monounsaturated to saturated fatty acids (MUFA:SFA ratio), red and processed meats, alcohol. The food group component of nuts was eliminated in our study because information on nuts consumption was not collected separately. For each of the components (except for alcohol), all participants were classified into quintiles according to their intake ranking. For fruits, vegetables, legumes, whole grains, fish, MUFA:SFA ratio, participants in the highest quintile received a score of 5, and those in the lowest quintile received a score of 1. For red and processed meats, participants in the highest quintile received a score of 1, and those in the lowest quintile received a score of 5. For alcohol, we classified individuals into five groups according to their alcohol consumption: (10,30], (0,10] or (30,40], 0 or (40,45], (45,50], and >50 grams per day for men; (5,15], (0,5] or (15,25], 0 or (25,30], (30,35], and >35 grams per day for women. Then, we assigned scores of 5, 4, 3, 2, 1 to corresponding individuals, respectively. The total score ranges from 8 to 40, and higher values of the score indicated greater adherence to AMED.

The DASH score was developed to measure adherence to a DASH-style diet. Due to the low consumption of nonfat and low-fat dairy products in our study population, a modified DASH score with nonfat and low-fat dairy replaced by full-fat dairy products [26] was adopted in our study. Additionally, we eliminated the food group component of sweetened beverages because the regular consumption of sweetened beverages was extremely low in our study population. As with the AMED score, the food group component of nuts was excluded. We constructed the DASH score based on quintile rankings within the population of each component as well, focusing on 7 components: fruits, vegetables, legumes, whole grains, full-fat dairy products, sodium, red and processed meat. For the first 5 components, the lowest intake was assigned 1 point, and the highest intake was assigned 5 points. In contrast, for sodium, red and processed meat, reverse scoring for the quintile ranks is used. By summing the component scores, we obtained an overall DASH score for each participant, ranging from 7 (no adherence) to 35 (perfect adherence). A detailed scoring criterion for dietary patterns is given in Supplementary Table 1-3.

### Study outcome

The outcome of this study was MAFLD. All participants underwent abdominal ultrasound, anthropometry measurements and blood tests at the baseline survey. Abdominal ultrasound was performed to assess the presence of hepatic steatosis by qualified ultrasonographers. Weight and height were measured by trained health workers using standardized methods. BMI was calculated as weight (kg) divided by height (m) squared; the BMI cutoff value of 25 kg/m^2^ was used to define the nonobese and obese Asian population, which is a widely used cutoff established by the WHO [27]. Waist circumference was measured as the midpoint between the iliac crest and the lower costal margin with tape all around the body in the horizontal position, taking the average of two measurements. For blood pressure, trained health workers measured blood pressure three times using an Omron HEM-8711 blood pressure monitor after participants had rested in a seated position for at least 5 min. For blood tests, after overnight fasting for at least 12 hours, blood samples were taken and shipped via cold chain to regional central laboratories (Chengdu, Chongqing, Guiyang, and Kunming). Within 24 hours, glycated hemoglobin (HbA1c) was tested by an MQ-6000 glycated hemoglobin analyzer (Shanghai Medconn Biotechnology Corporation); the plasma glucose and lipid profiles were tested by an AU5800 automated chemistry analyzer (Beckman Coulter Commercial Enterprise).

According to the latest diagnostic criteria for MAFLD proposed by an international expert panel [4], MAFLD in our study was defined by evidence of hepatic steatosis on ultrasound together with diabetes, overweight/obesity (i.e., BMI ≥23 kg/m^2^ for Asians), or two or more of the following metabolic risk abnormalities: waist circumference ≥90 cm in men and ≥80 cm in women; blood pressure ≥130/85 mmHg; plasma triglycerides (TG) ≥1.7 mmol/l; plasma high-density lipoprotein-cholesterol (HDL-C) <1.0 mmol/l for men and <1.3 mmol/l for women; prediabetes (fasting plasma glucose 5.6-6.9 mmol/l or hemoglobin A1c 5.7-6.4%). Note that we eliminated two metabolic conditions from the original definition since the insulinemia test and the high-sensitivity C-reactive protein (hs-CRP) test was not carried out at the baseline survey.

### Covariates

A directed acyclic graph (DAG) was constructed representing the existing literature to select a minimally sufficient set of covariates to adjust for confounding, according to the protocol of “Evidence Synthesis for Constructing Directed Acyclic Graphs” (ESC-DAGs) [28]. The independent tests were further carried out to continuously modify the proposed DAG. Based on the DAG, the following variables were retained as confounders in our statistical models: age, sex, urbanicity, ethnicity, marital status, highest education attained, household income, profession, regular smoking, physical activity in metabolic equivalent tasks, total energy intake, regular intake of sweeten beverage, regular intake of dietary supplements, regular intake of spicy food, regular intake of pepper food, insomnia symptoms, depressive symptoms, anxiety symptoms, menopause status for women, family history of cardiometabolic diseases, and BMI. Information on these selected confounders was mainly collected at the baseline questionnaire. A more detailed description of the DAG has been added as Supplementary Methods and Supplementary Fig. 1.

### Statistical analyses

Mean values and percentages of baseline characteristics were calculated for MAFLD status and obese status at baseline, with adjustment for age and sex by direct standardization (using the entire study sample as the standard population) as appropriate.

We used logistic regression with the inverse probability of exposure weighting (IPEW) to examine associations between dietary patterns and the outcome of MAFLD, with the lowest quintile as the reference for each dietary pattern. When analyzing the DASH score, we further adjusted for alcohol intake because alcohol was not part of that score. Associations were quantified by odds ratios (ORs) with 95% confidence intervals (CIs). To test for trends, models were conducted using the median of each quintile as a continuous variable. We next performed these models separately for nonobese and obese individuals, and heterogeneity between nonobese and obese individuals was evaluated with the Cochran Q test and chi-square (*χ*^2^) test. Subsequently, to facilitate the interpretations of the association between each dietary pattern and MAFLD, we evaluated the influence of each of the dietary components on the MAFLD risk by alternately subtracting one component at a time from the original score for each dietary pattern [29]. Because stringent data cleaning and audio reviewing were conducted, missing values were generated from the outliers that could not be verified. For the missing values of food groups, we used the chain equations method [30] for multiple imputation (5 times imputation).

Furthermore, we carried out subgroup analyses by sex, age, regular smoking, alcohol intake, physical activity, urbanicity, and ethnic group. The chi-square (*χ*^2^) test was used to examine heterogeneity among different strata. We also performed several sensitivity analyses: (1) using 24 kg/m^2^ as the BMI cutoff value of lean or overweight/obese individuals in the definition of MAFLD and using 28 kg/m^2^ to distinguish obese from nonobese individuals according to the guidelines for prevention and control of overweight and obesity in Chinese adults [31]; (2) assessing the association with each score in participants without exclusion of self-reported physician-diagnosed chronic hepatitis/cirrhosis, diabetes, hypertension, hyperlipidemia, coronary heart disease, stroke or cancer to examine the magnitude of potential reverse causality [3]; adopting the logistic regression without IPEW; (4) performing analyses with the complete case instead of imputed data; (5) assessing the associations of two dietary patterns with NAFLD risks. All statistical analyses were carried out with the R Project for Statistical Computing version 4.0.2 (Vienna, Austria).

## Results

### Participant characteristics

In the entire sample, 62.6% were women, the mean age was 49.5±11.0 years, 42.1% were ethnic minorities and the mean BMI was 23.8±3.4 kg/m^2^. After exclusion of self-reported physician-diagnosed outcomes, the prevalence of MAFLD was 16.1%. A total of 44,061 individuals (66.2%) had a BMI < 25.0 kg/m^2,^ and 22,465 individuals (33.7%) were obese. Age- and sex-standardized baseline characteristics, stratified by MAFLD and obese status, are shown in Table 1. In general, individuals with MAFLD had higher BMI and less physical activity than those without MAFLD but had similar AMED and DASH scores. They also tended to have higher SES (household income and profession) levels, higher consumption of beverage, spicy and pepper food, and a higher likelihood of reporting a family history of cardiometabolic diseases. Similar characteristics were also found among nonobese and obese populations. Of note, compared to obese MAFLD, nonobese MAFLD individuals were more likely to be current smokers and had a higher consumption of alcohol.

**Table 1:** Age- and sex-standardized baseline characteristics in the CMEC study, according to obesity and MAFLD status

Characteristic	Overall ^**a**^ ***n*** = 66,526		Nonobese ^**b**^ ***n*** = 44,061		Obese ^**c**^ ***n*** = 22,465	
	No MAFLD ***n*** = 55,730	MAFLD ***n*** = 10,742	No MAFLD ***n*** = 41,310	MAFLD ***n*** = 2,720	No MAFLD ***n*** = 14,420	MAFLD ***n*** = 8,022
AMED score	*24.6±4.3*	*24.8±4.6*	*24.6±4.3*	*25.2±4.5*	*24.5±4.4*	*24.8±4.6*
DASH score	*20.6±4.3*	*20.5±4.5*	20.7±4.4	20.8±4.7	20.4±4.3	20.4±4.5
Age (year)	*49.5±11.1*	*50.0±10.2*	*49.5±11.3*	*51.0±10.3*	49.5±10.3	49.6±10.2
Female sex (%)	*64.6*	*51.6*	*65.5*	*54.8*	62.1	50.6
Urban residence (%)	*32.9*	*42.7*	*32.9*	*42.1*	*32.7*	*43.0*
Ethnic minorities (%)	*42.6*	*40.6*	*41.4*	*33.8*	*46.0*	*42.8*
Married or cohabiting (%)	89.9	90.1	89.8	90.6	*90.2*	*90.0*
Highest education completed (%)						
No formal school	25.3	24.4	*24.2*	*19.4*	*28.3*	*26.1*
Primary school	24.9	25.6	*25.0*	*24.8*	*24.7*	*25.8*
Middle and high school	38.0	37.9	*38.5*	*41.9*	*36.8*	*36.5*
College or university	11.8	12.1	*12.3*	*13.9*	*10.1*	*11.6*
Household income (Yuan/year) (%)						
<12000	*16.6*	*14.7*	*16.9*	*13.9*	*15.4*	*15.0*
12000-19999	*18.3*	*17.6*	*18.1*	*16.5*	*18.9*	*17.9*
20000-59999	*37.1*	*36.5*	*36.9*	*36.1*	*37.9*	*36.7*
60000-99999	*14.5*	*15.9*	*14.5*	*16.1*	*14.7*	*15.7*
100000-199999	*10.8*	*12.1*	*10.7*	*13.9*	*10.9*	*11.5*
>200000	*2.7*	*3.2*	*2.8*	*3.6*	*2.2*	*3.2*
Occupation (%) ^d^						
Primary industry practitioner	*36.5*	*30.3*	*38.1*	*30*	*32.2*	*30.2*
Secondary industry practitioner	*8.2*	*6.8*	*8.2*	*6.4*	*8.1*	*7.0*
Tertiary industry practitioner	*37.5*	*41.2*	*36.5*	*42.7*	*39.8*	*40.8*
Unemployed	*17.8*	*21.6*	*17.1*	*20.9*	*20.0*	*21.9*
Regular smoking (%)						
Never	*76.2*	*77.2*	75.3	75.4	*78.6*	*77.9*
Previous	*3.8*	*4.6*	3.5	4.0	*4.7*	*4.9*
Current	*20.0*	*18.2*	21.2	20.7	*16.7*	*17.3*
Alcohol intake ^e^						
None	56.5	57.1	*57.1*	*56.6*	*54.7*	*57.2*
Low	37.2	36.5	*36.5*	*35.7*	*39.2*	*36.9*
High	6.3	6.4	*6.4*	*7.7*	*6.1*	*5.9*
Physical activity (MET-h/day)	*26.4±17.0*	*23.8±16.8*	*26.8±17.1*	*23.7±16.0*	*25.4±16.8*	*23.8±17.2*
BMI (kg/m^2^)	*23.2±3.0*	*27.2±3.4*	*21.8±2.0*	*23.7±1.2*	*27.1±1.9*	*28.4±2.9*
Dietary supplement (%)	*15.5*	*13.9*	16.4	16.5	*12.7*	*12.9*
Regular beverage intake (%)						
Never	*92.7*	*90.2*	93.4	93.5	*90.9*	*89.0*
Previous	*0.4*	*0.4*	0.4	0.4	*0.5*	*0.4*
Current	*6.9*	*9.5*	6.3	6.0	*8.6*	*10.6*
Regular spicy food intake (%)	*79.1*	*81.2*	*79.4*	*83.1*	*78.7*	*80.5*
Regular pepper food intake (%)	*67.4*	*69.7*	*67.8*	*73.8*	*66.6*	*68.2*
Insomnia Symptoms (%)	*42.1*	*40.4*	*42.3*	*38.8*	*41.6*	*41.0*
Depressive symptom (%)	*4.6*	*3.4*	*4.7*	*3.7*	*4.3*	*3.4*
Anxiety symptom (%)	*5.5*	*4.3*	*5.5*	*3.8*	*5.4*	*4.5*
Menopausal status in women (%)						
Premenopause	*52.8*	*50.5*	*52.3*	*49.0*	*54.0*	*51.1*
Perimenopause	*7.0*	*7.8*	*6.9*	*7.3*	*7.4*	*8.0*
Postmenopause	*40.2*	*41.7*	*40.8*	*43.7*	*38.6*	*40.9*
Family history of cardiometabolic disease (%)	*31.0*	*34.8*	*30.7*	*36.4*	*32.0*	*34.2*

Data are presented as the mean (± SD) or percentage

*CMEC* the China Multi-Ethnic Cohort Study, *MAFLD* metabolic dysfunction-associated fatty liver disease, *AMED* Alternate Mediterranean diet, *DASH* Dietary Approaches to Stop Hypertension

Linear regression analyses and chi-square tests were used to compare different groups for continuous variables and categorical variables, respectively. If the difference across MAFLD status (No MAFLD, MAFLD) was significant (*p* < 0.05), values were shown in *italic*

^a^ There were 54 missing values of MAFLD status in the total population

^b^ There were 31 missing values of MAFLD status in the nonobese population

^c^ There were 23 missing values of MAFLD status in the obese population

^d^ Primary industry practitioners are defined as farming, forestry, animal husbandry and fishery laborer. Secondary industry practitioners refer to workers in the processing and manufacturing industry. Tertiary industry practitioners refer to workers in industries other than primary and secondary industries

^e^ Low alcohol intake is defined as less than 140 g/day for men and less than 70 g/day for women; high alcohol intake is defined as more than 140 g/day for men and more than 70 g/day for women

### Dietary patterns and MAFLD

The adjusted association of AMED and DASH with MAFLD is shown in Table 2. There was a significant inverse trend with increasing DASH scores and MAFLD risks (*P*_*trend*_ < 0.001). Compared with participants in the lowest quintile of the DASH score, those in the highest quintile showed strong inverse associations with risks of MAFLD (OR = 0.85; 95% CI, 0.80-0.91) after controlling for potential confounders. However, there seemed to be a null association between AMED (OR = 0.97; 95% CI, 0.91-1.04; *P*_*trend*_ = 0.361) and MAFLD risk.

**Table 2:** Adjusted associations between AMED and DASH and MAFLD in total, nonobese and obese populations

	Overall MAFLD		Nonobese MAFLD		Obese MAFLD		Heterogeneity test ^**b**^
	No. of case	OR (95% CI) ^**a**^	No. of case	OR (95% CI) ^**a**^	No. of case	OR (95% CI) ^**a**^	
**AMED**							
Quintile 1	1,863	1 (Reference)	377	1 (Reference)	1486	1 (Reference)	*I*^*2*^= 7.4%; *P*= 0.365
Quintile 2	2,301	1.01(0.95,1.08)	549	0.98(0.87,1.11)	1107	1.03(0.94,1.12)	
Quintile 3	1,824	0.98(0.92,1.05)	494	1.03(0.91,1.16)	1975	0.97(0.89,1.06)	
Quintile 4	1,802	1.01(0.94,1.07)	491	1.04(0.92,1.18)	1311	0.99(0.91,1.08)	
Quintile 5	2,905	0.97(0.91,1.04)	795	1.03(0.91,1.16)	2110	0.93(0.86,1.02)	
*P* trend		0.361		0.432		0.073	
**DASH**							
Quintile 1	2,011	1 (Reference)	493	1 (Reference)	1518	1 (Reference)	*I*^*2*^= 78.5%; *P*= 0.001
Quintile 2	1,551	0.98(0.92,1.04)	564	0.82(0.73,0.92)	1210	1.07(0.98,1.16)	
Quintile 3	2,821	0.96(0.90,1.02)	477	0.82(0.73,0.92)	1421	1.03(0.95,1.12)	
Quintile 4	1,780	0.94(0.88,1.00)	649	0.79(0.70,0.89)	2002	0.97(0.89,1.06)	
Quintile 5	2,532	0.85(0.80,0.91)	523	0.69(0.61,0.78)	1838	0.90(0.83,0.98)	
*P* trend		< 0.001		< 0.001		0.002	

*MAFLD* metabolic dysfunction-associated fatty liver disease, *AMED* Alternate Mediterranean diet, *DASH* Dietary Approaches to Stop Hypertension, *OR* odds ratio, *CI* confidence interval

^a^ Logistic regression models with the inverse probability of exposure weighting were adjusted for: age, sex, urbanicity, ethnicity, marital status, highest education attained, household income, profession, regular smoking, physical activity in metabolic equivalent tasks, total energy intake, regular intake of sweeten beverage, regular intake of dietary supplements, regular intake of spicy food, regular intake of pepper food, insomnia symptoms, depressive symptoms, anxiety symptoms, menopause status for women, family history of cardiometabolic diseases, and BMI. Models for DASH were additionally adjusted for alcohol intake.

^b^ Heterogeneity tests were performed between nonobese MAFLD and obese MAFLD.

Table 2 also shows the associations of the two dietary patterns with MAFLD stratified by obese status. We observed similar results in nonobese and obese populations as in the total population. Moreover, DASH showed stronger (*I*^2^ = 78.5 % ; *P*_*heterogeneity*_ = 0.001) associations with nonobese MAFLD (OR = 0.69; 95% CI, 0.61-0.78; *P*_*trend*_ < 0.001) than with obese MAFLD (OR = 0.90; 95% CI, 0.83-0.98; *P*_*trend*_ < 0.001). AMED was not associated with nonobese MAFLD or obese MAFLD, with no differences in obese status (*I*^2^ = 7.4 % ; *P*_*heterogeneity*_ = 0.365).

### Dietary components analyses

Because the trends of associations were similar among different populations, only the contribution of each component of AMED and DASH to MAFLD risks in the total population was further examined, as shown in Table 3. For AMED, a weak inverse association with MAFLD (OR = 0.95; 95% CI, 0.90-0.99) was shown after excluding the MUFA:SFA ratio. Because the OR value of AMED overall was very close to 1, the proportions of contributions seemed quite large. For DASH, full-fat dairy products and sodium contributed predominantly to the positive associations. The association with MAFLD risk was attenuated after excluding full-fat dairy products (OR = 0.93; 95% CI, 0.89-0.97), sodium (OR = 0.89; 95% CI, 0.86-0.93), and the proportions of contributions to the associations were 50.6% and 20.5%, respectively.

**Table 3:** Odds ratios associated with AMED and DASH scores and after alternate subtraction of each of its dietary components in the total population

Dietary variable	OR (95% CI) ^**a**^	***P*** value	Reduction in apparent association (%) ^**b**^
**AMED**			
AMED overall	0.99(0.94,1.03)	0.551	-
AMED minus vegetables	1.01(0.96,1.06)	0.722	42.2
AMED minus legumes	1.01(0.96,1.06)	0.784	54.2
AMED minus fruits	0.99(0.94,1.04)	0.655	26.6
AMED minus whole grains	0.99(0.95,1.04)	0.735	43.9
AMED minus red & processed meat	1.02(0.97,1.07)	0.363	-54.7
AMED minus fish	0.96(0.92,1.01)	0.094	-167.7
AMED minus alcohol	0.99(0.95,1.03)	0.549	10.5
AMED minus MUFA: SFA ratio	0.95(0.90,0.99)	0.017	-275.4
**DASH**			
DASH overall	0.86(0.83-0.90)	< 0.001	-
DASH minus vegetables	0.88(0.84-0.91)	< 0.001	11.5
DASH minus legumes	0.87(0.83-0.90)	< 0.001	3.6
DASH minus fruits	0.85(0.82-0.89)	< 0.001	-6.5
DASH minus whole grains	0.85(0.82-0.89)	< 0.001	-7.7
DASH minus full-fat dairy products	0.93(0.89-0.97)	0.001	50.6
DASH minus sodium	0.89(0.86-0.93)	< 0.001	20.5
DASH minus red & processed meat	0.88(0.85-0.92)	< 0.001	15.8

*MUFA* monounsaturated fatty acid, *SFA* saturated fatty acid, *AMED* Alternate Mediterranean diet, *DASH* Dietary Approaches to Stop Hypertension, *OR* odds ratio, *CI* confidence interval

^a^ We assumed a linear relationship with ORs representing the risk change per 25% score range increment

^b^ Reduction in apparent effect (%) = (OR_AMED/DASH overall_ - OR_AMED/DASH minus component_)/(OR_AMED/DASH overall_ - 1)*100%

### Subgroup and sensitivity analyses

**Fig. 2:**
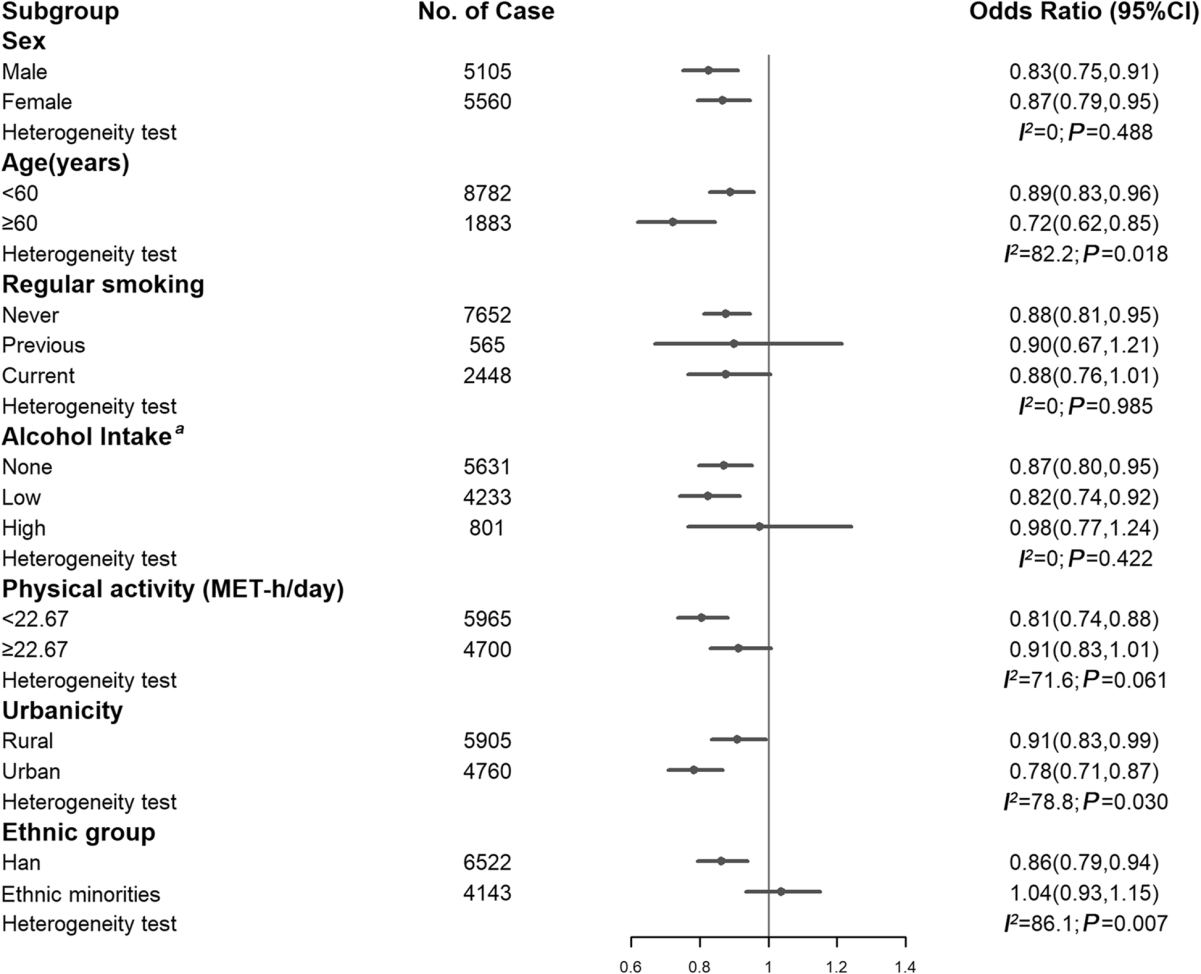
Subgroup analysis of estimated associations between DASH and MAFLD in the total population. CI, confidence interval; MET, metabolic equivalent task. ^a^ Low alcohol intake is defined as less than 140 g/day for men and less than 70 g/day for women; high alcohol intake is defined as more than 140 g/day for men and more than 70 g/day for women

Because of similar association trends among different populations, subgroup analyses were only constructed between DASH and MAFLD in the total population. As shown in Figure 2, the associations of DASH and MAFLD were consistent across participants, irrespective of sex, smoking status, physical activity level, alcohol consumption, and ethnic groups (all *P*_*heterogeneity*_ > 0.05). Significant differences across strata were observed for age, urbanicity, and ethnic groups. Compared to the ethnic minorities, DASH showed a stronger association with MAFLD among the Han majority, which was agreed with the stratified results of urbanicity because nearly all of the ethnic minorities lived in rural areas.

Overall, sensitivity analyses (e.g., applying the Chinese standard definition of overweight and obesity, without exclusion of individuals with self-reported physician-diagnosed diseases, using logistic regression) provided similar results and are detailed in Supplementary Tables 4–6. When performing the analysis with the complete cases, similar results were found, albeit the heterogeneity of DASH between nonobese and obese MAFLD was slightly attenuated and statistically not significant (Supplementary Table 7). When using NAFLD as the outcome, we found that the associations of dietary patterns and NAFLD were similar to results of MAFLD, but compared to MAFLD definition, the prevalence of NAFLD was lower (15.4%) and less obese individuals with fatty liver were identified (Supplementary Table 8).

## Discussion

To the best of our knowledge, this is the first large population-based study to examine whether Mediterranean or DASH is associated with MAFLD in LEMRs. This study shows that better adherence to DASH but not the AMED dietary pattern was significantly associated with a lower risk of MAFLD. The association was more pronounced in nonobese MAFLD participants than in obese MAFLD participants. Furthermore, our study indicated that the same dietary guidelines should be recommended to MAFLD individuals as NAFLD individuals in LEMRs.

While the term MAFLD has only been recently introduced to replace NAFLD [4], there are strong indications of acceptance and endorsement worldwide for MAFLD [32–35]. To date, a few studies have reported that compared with the NAFLD criteria, the MAFLD criteria could identify a greater number of individuals with metabolically complicated fatty liver [36–38]. Consistent with these studies, we found that the change from NAFLD to MAFLD could identify more fatty liver individuals with obesity in our study. Though we confirmed that the same dietary guidelines should be recommended to both NAFLD and MAFLD, individuals with poor metabolic health may benefit from the definition shift when it comes to disease prevention.

To our knowledge, only few studies have examined the effects of dietary patterns and NAFLD in less-developed regions. Using a factor analysis, which is an a posteriori, data-driven method to define dietary patterns, a cohort study in Tianjin of China identified 3 dietary patterns: sugar rich dietary pattern, vegetable rich dietary pattern, and animal food dietary pattern [39]. Dietary patterns rich in animal foods or sugar were associated with an increased risk of NAFLD, whereas a vegetable rich dietary pattern was not associated [39]. A case-control study [40] and a cross-sectional study [41] from China have also shown a positive association between animal foods pattern and NAFLD. Another cross-sectional study with 1,639 participants in Shandong of China identified three a posteriori patterns, traditional Chinese, Western, and high-energy dietary patterns [42]. This study found that adherence to the western dietary pattern was associated with higher risks of NAFLD [42]. A cross-sectional study with 797 adults in China has reported that Diet Quality Index-International but not the Mediterranean diet was associated with lower prevalence of NAFLD [43]. These studies generally strengthened the importance of a healthy dietary pattern in preventing NAFLD development or progression in less-developed regions, but the evidence considering ethnic disparities was lacking. In this study, based on a larger-scale population and focusing on areas with diverse ethnic minorities, we found it necessary to generalize a healthy dietary pattern like DASH to LEMRs.

Our findings of DASH with MAFLD agree with previous studies investigating the association of DASH with NAFLD [44, 45]. In a randomized controlled clinical trial addressing weight loss and metabolic status among NAFLD participants from Iran, consumption of a DASH diet for 8 weeks had a beneficial effect on body weight and metabolic profiles, including liver enzymes, triglycerides, insulin metabolism markers, inflammatory markers, and oxidative stress markers [44]. However, all of the participants in that study were either overweight or obese. Recently, another Chinese study showed that adherence to the DASH diet was independently associated with a markedly lower prevalence of NAFLD in middle-aged and elderly adults [45], but BMI was not taken into account as a covariate or a stratification factor but a mediator in this study. Our study confirmed and refined these associations, and we focused on the phenotype of MAFLD, especially nonobese MAFLD, showing that the protective effect of DASH on nonobese MAFLD was greater than that of obese MAFLD.

In our study, we found that full-fat dairy products and sodium contributed predominantly to the association of DASH with MAFLD. Although studies linking dairy products and sodium to NAFLD are scarce, there is strong evidence regarding associations between the two components and metabolic disorders, which supports our findings. For dairy products, findings from the Prospective Urban Rural Epidemiology (PURE) study [46] and another Chinese cohort study [47] demonstrated that dairy consumption was associated with a significantly lower risk of diabetes and favorable changes in cardiometabolic traits, indicating that consumption of dairy products should be encouraged in less-developed countries where dairy consumption is low. Some nutrients, such as milkfat, vitamin D, calcium, magnesium, potassium, and whey proteins, in dairy products may play protective roles [48]. For instance, calcium may interact with SFAs in milkfat to decrease fat absorption, lower TG concentrations, and improve the HDL-C to low-density lipoprotein cholesterol (LDL-C) ratio [49, 50]. For sodium, a significant positive association with the risk of NAFLD and metabolic syndrome was demonstrated in recent studies [51–53]. It has been proposed that high sodium intake might impair the insulin signaling step, leading to dysregulation of insulin sensitivity and increased fat buildup in the body [54]. For decades, a high level of sodium intake has remained the leading dietary risk factor for metabolic disorders in China [55]. As a dietary pattern initially developed to control sodium intake, it is not surprising that there was a consistently inverse association for DASH with the risk of MAFLD in our study.

Previous studies from European countries showed greater adherence to the Mediterranean diet was associated with lower prevalence of NAFLD [56, 57], and a Mediterranean-style diet has been recommended for the management of NAFLD by the EASL-EASD-EASO Clinical Practice Guideline [13]. However, the effects of Mediterranean-style diets on NAFLD or related cardiometabolic risks are still controversial, especially in non-Mediterranean regions [58, 59]. Contrary to findings in European countries, our results did not find a significant association between AMED and MAFLD, which was consistent with another Chinese cross-sectional study of NAFLD [43]. In our study, the MUFA:SFA ratio appeared to be the most influential component that was positively associated with MAFLD. This may be related to a serious lack of high-quality sources of MUFAs (such as olive oil or marine fish) and the deep cooking manner in less-developed regions, which are quite different from Mediterranean regions.

Diet modification guidelines for individuals with normal body weight have been an unresolved issue in real-world clinical practice. To date, the importance of BMI has been demonstrated in many diet studies on hepatic steatosis [10, 56, 60]. However, only a few epidemiological studies have specifically investigated the role of diet between nonobese NAFLD and obese NAFLD, but they all focused on dietary macronutrient composition, such as protein [10] and carbohydrate [60]. Our results added to the limited number of studies that evaluated the association between diet and MAFLD in nonobese and obese individuals and showed the importance of dietary patterns beyond the single nutrition components in nonobese MAFLD individuals, which can facilitate adoption in both public health and clinical practice. In our study, we confirmed that both obese MAFLD and nonobese MAFLD individuals could follow a DASH diet, and the association was more pronounced in nonobese MAFLD individuals.

The reason why DASH showed a more pronounced relationship with nonobese MAFLD is unclear. Based on the evidence of NAFLD, one explanation is that participants with nonobese MAFLD could have different genetic predispositions from obese MAFLD, as with NAFLD. Patatin-like phospholipase domain-containing 3 (PNPLA3) is one of the most important genetic determinants of NAFLD [61], and the PNPLA3 rs738409 gene polymorphism is strongly associated with hepatic fat levels and hepatic inflammation [61]. Carriers of the PNPLA3 rs738409 G allele experience greater improvement in liver fat after lifestyle modification, with a greater reduction in blood total cholesterol, LDL-C level [62]. As a population study reported, the G allele at PNPLA3 rs738409 was more common in nonobese NAFLD participants than in obese NAFLD participants [63]. The difference in the G allele may partly explain the heterogeneity in our results between nonobese and obese individuals.

The main strength of this study is the large-scale population from LEMRs, providing a unique opportunity to examine the associations between two a priori dietary patterns (i.e., AMED and DASH) and MAFLD risks, particularly the associations with nonobese MAFLD risks, in less-developed regions where nonobese MAFLD has been an increasingly concerning health issue. Furthermore, we performed analyses with the application of ESC-DAGs and IPEW, which are under the framework of causal inference.

Nonetheless, some limitations need to be addressed. First, the FFQ used in our study only included 13 crude food groups, not specific food items. The tool might be flawed, but considering that most participants from our study regions speak different local languages that need to be translated from the local translators, and consume local foods that are not found in any existing food database, the food group-based and simplified food questionnaire may be the only feasible way to collect dietary information from various ethnic regions in a large population-based study. However, it should not have a significant impact on the assessment of dietary patterns and total energy intake. By analyzing the subsample data of 24HDR, we found that the differences between the calculation of total energy intake based on crude food groups and that based on specific food items were small and roughly randomly distributed around zero. Second, although we used a validated FFQ to measure dietary intake, recall bias could not be easily ruled out. Third, as most of the current measurement error correction methods are only applicable to a single food item or nutrient [64, 65], we did not correct the measurement errors in this study. Forth, in the assessment of MAFLD, the objective indicators of insulin and hs-CRP were not available in our baseline survey for the sake of feasibility. Fifth, whereas we adjusted for a variety of confounders based on a DAG, there might still be risk of residual confounding. Sixth, our study population only consisted of Chinese individuals, which may also limit the generalizability of our findings to other LEMRs from other parts of the world.

## Conclusion

In conclusion, we found that a DASH diet was associated with MAFLD in LEMRs, and this relationship appeared to be more pronounced in nonobese MAFLD individuals than in obese MAFLD individuals. The results of this large population-based study add to the current evidence on the importance of dietary patterns in MAFLD, suggesting that DASH is superior dietary guidance to AMED regarding reducing MAFLD risks in LEMRs, especially in nonobese individuals with MAFLD.

## Supplementary Information

**Additional file 1: Supplementary Text 1.** Diet-related questionnaires (excerpt from the CMEC questionnaires). **Supplementary Table 1.** Scoring criteria for the AMED score in the CMEC study. **Supplementary Table 2.** Scoring criteria for the DASH score in the CMEC study. **Supplementary Table 3.** Exchange values of fatty acids for food groups included in the CMEC study^a^. **Supplementary Methods**: directed acyclic graphs (DAG). **Supplementary Figure 1.** The final constructed DAG. **Supplementary Table 4.** Estimated associations using 24 kg/m^2^ and 28 kg/m^2^ as the BMI cutoff value of lean or overweight or obese individuals. **Supplementary Table 5.** Estimated associations in participants without exclusion of self-reported physician diagnosed chronic hepatitis/cirrhosis, diabetes, hypertension, hyperlipidemia, coronary heart disease, stroke or cancer. **Supplementary Table 6.** Estimated associations using logistic regression. **Supplementary Table 7.** Estimated associations with the complete case instead of imputed data. **Supplementary Table 8.** Adjusted associations between AMED and DASH and NAFLD.

## Data Availability

Data described in the manuscript, codebook, and analytic code will be available from the corresponding author under reasonable request. (Xing Zhao, Ph.D, email: xingzhao@scu.edu.cn)

## Abbreviations

MAFLD: Metabolic dysfunction-associated fatty liver disease
SES: Socioeconomic status
DASH: Dietary Approaches to Stop Hypertension
LEMRs: Less-developed ethnic minority regions
BMI: Body mass index
CMEC: The China Multi-Ethnic Cohort Study
AMED: Alternate Mediterranean diet
FFQ: Food frequency questionnaire
24HDR: 24-hour dietary recall
DAG: Directed acyclic graph
OR: Odds ratio
CI: Confidence interval
NAFLD: Non-alcoholic fatty liver disease
MUFA:SFA ratio: ratio of monounsaturated to saturated fatty acids
