# Supplementary material for: Healthy dietary patterns and metabolic dysfunction-associated fatty liver disease in less-developed ethnic minority regions: a large cross-sectional study
Source: BMC Public Health. 2022 Jan 17;22:118. doi: 10.1186/s12889-021-12486-x (PMC8764858; doi:10.1186/s12889-021-12486-x)
Supplement: Supplementary file 1 — Additional file 1: Supplementary Text 1. Diet-related questionnaires (excerpt from the CMEC questionnaires). Supplementary Table 1. Scoring criteria for the AMED score in the CMEC study. Supplementary Table 2. Scoring criteria for the DASH score in the CMEC study. Supplementary Table 3. Exchange values of fatty acids for food groups included in the CMEC studya. Supplementary Methods: directed acyclic graphs (DAG). Supplementary Figure 1. The final constructed DAG. Supplementary Table 4. Estimated associations using 24 kg/m2 and 28 kg/m2 as the BMI cutoff value of lean or overweight or obese individuals. Supplementary Table 5. Estimated associations in participants without exclusion of self-reported physician diagnosed chronic hepatitis/cirrhosis, diabetes, hypertension, hyperlipidemia, coronary heart disease, stroke or cancer. Supplementary Table 6. Estimated associations using logistic regression. Supplementary Table 7. Estimated associations with the complete case instead of imputed data. Supplementary Table 8. Adjusted associations between AMED and DASH and NAFLD. [file 12889_2021_12486_MOESM1_ESM.docx]

**Supplementary material**

**Article title**

Healthy dietary patterns and metabolic dysfunction-associated fatty liver disease in less-developed ethnic minority regions: a large cross-sectional study

**Journal title**

BMC public health

**Authors**

Xiaofen Xie^1*^, Bing Guo^1^^*^, Xiong Xiao^1^, Jianzhong Yin^2,3^, Ziyun Wang^4^, Xiaoman Jiang^5^, Jingzhong Li^6^, Lu Long^1^, Junmin Zhou^1^, Ning Zhang^1^, Yuan Zhang^1^, Ting Chen^7**^, Baima Kangzhuo^8**^, and Xing Zhao^1**^

^*^ These authors contributed equally as first co-authors.

^**^ These authors contributed equally as senior co-authors.

**Author affiliations**

^1^West China School of Public Health and West China Fourth Hospital, Sichuan University, Chengdu, China; ^2^School of Public Health, Kunming Medical University, Kunming, China; ^3^Baoshan College of Traditional Chinese Medicine, Kunming, China; ^4^School of Public Health, the key Laboratory of Environmental Pollution Monitoring and Disease Control, Ministry of Education, Guizhou Medical University, Guiyang, China; ^5^Chengdu Center for Disease Control and Prevention, Chengdu, China; ^6^Tibet Center for Disease Control and Prevention, Lhasa, China; ^7^Chongqing Municipal Center for Disease Control and Prevention, Chongqing, China; ^8^Tibet University, Lhasa, China.

**Correspondence**

Xing Zhao, Ph.D.

Department of Epidemiology and Biostatistics

West China School of Public Health and West China Fourth Hospital, Sichuan University

No.16, People's South Road, Chengdu, Sichuan, China, 610041

E-mail: [xingzhao@scu.edu.cn](mailto:xingzhao@scu.edu.cn)

Or

Baima Kangzhuo, Ph.D.

Tibet University

No.10, East Tibet University Road, Lhasa, China 850000

E-mail: [bmkz123@163.com](mailto:bmkz123@163.com)

Or

Ting Chen, M.Sc.

Chongqing Municipal Center for Disease Control and Prevention

No.8, Changjiang 2nd Road, Chongqin, China, 400042

E-mail: [640963985@qq.com](mailto:640963985@qq.com)

Table of contents

[Supplementary Text 1. Diet-related questionnaires (excerpt from the CMEC questionnaires) 4](#_Toc88072412)

[Supplementary Table 1-3. The scoring criterion for dietary patterns. 8](#_Toc88072413)

[Supplementary Table 1. Scoring criteria for the AMED score in the CMEC study. 8](#_Toc88072414)

[Supplementary Table 2. Scoring criteria for the DASH score in the CMEC study. 9](#_Toc88072415)

[Supplementary Table 3. Exchange values of fatty acids for food groups included in the CMEC study ^a^ 10](#_Toc88072416)

[Supplementary Methods: directed acyclic graphs (DAG) 11](#_Toc88072417)

[Supplementary Figure 1: The final constructed DAG. 12](#_Toc88072418)

[Supplementary Table 4-8: Sensitivity analyses 13](#_Toc88072419)

[Supplementary Table 4: Estimated associations using 24 kg/m^2^ and 28 kg/m^2^ as the BMI cutoff value of lean or overweight or obese individuals. 13](#_Toc88072420)

[Supplementary Table 5: Estimated associations in participants without exclusion of self-reported physician diagnosed chronic hepatitis/cirrhosis, diabetes, hypertension, hyperlipidemia, coronary heart disease, stroke or cancer. 14](#_Toc88072421)

[Supplementary Table 6: Estimated associations using logistic regression. 15](#_Toc88072422)

[Supplementary Table 7: Estimated associations with the complete case instead of imputed data. 16](#_Toc88072423)

[Supplementary Table 8: Adjusted associations between AMED and DASH and NAFLD. 17](#_Toc88072424)

# Supplementary Text 1. Diet-related questionnaires (excerpt from the CMEC questionnaires)

| **Alcohol consumption** | | | | |
| --- | --- | --- | --- | --- |
| **A1 Have you drunk any alcohol during past 24 hours？**  □ Yes □ No | | | | |
| **A2 During the past 12 months, how often did you drink any alcohol？** | | | | |
| □ Never | | | | → jump to next section |
| □ Only occasionally  □ Only at certain seasons  □ Every week but less than weekly | | | | |
| □ At least once a week | | | | → jump to A4 |
| **A3a In the past, have you ever drunk every week for at least one year？** | | | | |
| □ Yes | | | | → jump to A3b |
| □ No | | | | → jump to next section |
| **A3b How long have you stopped this behavior (drunk every week for at least one year) ？**  □ □ years → jump to next section | | | | |
| **A4 During the past 12 months, on how many days did you drink alcohol in a typical week？**  □ 1-2 days/week  □ 3-5 days/week  □ Daily/almost everyday | | | | |
| **A5 When did you start drinking some alcohol in most weeks?**  □ years old | | | | |
| **A6 On three different situations, what kind(s) of alcoholic drinks you choose and how much you usually drink in a day？（Can choose up to 3 types of alcohol for special occasions）** | | | | |
|  | Typical occasion (choose one) | On a special day when you drink a lot | Last time when you drink | |
| Beer | □ □ Bottle/week | □ □ Bottle/day | □□Bottle/day | |
| rice wine /fruit wine（<10°） | □ □ ^*^liang/week | □ □ ^*^liang /day | □ □ ^*^liang /day | |
| Highland barley wine | □ □ ^*^liang /week | □ □ ^*^liang /day | □ □ ^*^liang /day | |
| Wine | □ □ ^*^liang /week | □ □ ^*^liang /day | □ □ ^*^liang /day | |
| Spirit or Chinese baijiu（≥40°） | □ □ ^*^liang /week | □ □ ^*^liang /day | □ □ ^*^liang /day | |
| Spirits or Chinese baijiu (<40°) | □ □ ^*^liang /week | □ □ ^*^liang /day | □ □ ^*^liang /day | |
| Rice wine (30°-40°） | □ □ ^*^liang /week | □ □ ^*^liang /day | □ □ ^*^liang /day | |
| * liang ：This is one of the mass units being used in modern China. 1 liang = 50 g | | | | |

**A7 On a typical day when you drink alcohol, when do you usually take the drink？**

□ Usually before meals

□ Drink with meals

□ Usually drink between or after meals

□ Usually before sleep

□ No regular pattern

**A8 After drinking alcohol, do you usually experience hot flushes or dizziness？**

□ Yes, soon after first mouthful

□ Yes, after drinking small amount of alcohol

□ Yes, but only after drinking large amount of alcohol

□ No

**A9 During the past month, have you ever had the following experience？**

|  | **Yes** | **No** |
| --- | --- | --- |
| Unable to work or to do anything because of drinking | □ | □ |
| Felt depressed, angry or couldn't control yourself after drinking | □ | □ |
| Could not keep away from drinking | □ | □ |
| Had shakes when you stopped drinking | □ | □ |

**A10 Has your alcohol consumption changed significantly compared with that some years ago？**

□ About same as before

□ Increased significantly

□ Decreased significantly

| **Tea and Beverages** | | | | | | | | | | | | | | | | |  |  |
| --- | --- | --- | --- | --- | --- | --- | --- | --- | --- | --- | --- | --- | --- | --- | --- | --- | --- | --- |
| **1. Tea** | | | | |  | | | | | | |  |  | | | |  |  |
| **B1** | **Have you ever drunk tea weekly and last over half year？** | | | | | | | | | | | | | | | |  |  |
| □ Yes | | | | |  | | | | | | |  |  | | | |  |  |
| □ No → jump to B9 | | | | | | | | | | | | | | | | |  |  |
| **B2** | **When did you start drinking tea in most weeks？**  **□** years old | | | | | | | | | | | | | | | |  |  |
| **B3** | **During the past 12 months, how many days did you drink tea in a typical week？**  □ Don not drink tea currently → jump to B3a since □ □ year old stop drinking | | | | | | | | | | | | | | | |  |  |
| □ 1-2 day(s)/week | | | | |  | | | | | | |  |  | | | |  |  |
| □ 3-5 days/week | | | | |  | | | | | | |  |  | | | |  |  |
| □ Daily/almost everyday | | | | |  | | | | | | |  |  | | | |  |  |
| **B4** | **On days when you drink, which type of tea do you prefer, how many cups do you usually drink？（choose one only）** | | | | | | | | | | | | | | | |  |  |
| Green tea | | | | | □ □ cups/day | | | | | | |  |  | | | |  |  |
| Jasmine | | | | | □ □ cups/day | | | | | | |  |  | | | |  |  |
| Dark tea (brick tea, Pu-erh tea, etc) | | | | | □ □ cups/day | | | | | | |  |  | | | |  |  |
| Sweety tea (milk+ black tea) | | | | | □ □ cups/day | | | | | | |  |  | | | |  |  |
| Black tea | | | | | □ □ cups/day | | | | | | |  |  | | | |  |  |
| Oolong tea (Tieguanyin tea) | | | | | □ □ cups/day | | | | | | |  |  | | | |  |  |
| Yellow tea (Mengdinghuaya tea) | | | | | □ □ cups/day | | | | | | |  |  | | | |  |  |
| White tea (Yinzhen tea) | | | | | □ □ cups/day | | | | | | |  |  | | | |  |  |
| **B5** | **How often do you change tea leaves during a day?** | | | | | | | | | | |  | □ □ times | | | |  |  |
| **B6** | **How much tea leaves do you usually add each time？** | | | | | | | | | | □ □ grams | | | | | |  |  |
| **B7** | **What strength of tea do you usually prefer to drink？**  □ Weak □ Moderate □ Strong | | | | | | | | | | | | | | | |  |  |
| **B8** | **What temperature do you usually drink your tea？**  □ Bunning hot □ Hot □ Room temperature / warm | | | | | | | | | | | | | | | |  |  |
| **2. Beverages** | | | | |  | | | | | | |  |  | | | |  |  |
| **B9** | **Have you ever drunk every week and last over half year？** | | | | | | | | | | | | | | | |  |  |
| □ Yes | | | | |  | | | | | | |  |  | | | |  |  |
| □ No → jump to next section | | | | | | | | | | | | | | | | |  |  |
| **B10** | **How old did you start to develop the habit of drinking beverages？**  □ □ years old | | | | | | | | | | | | | | | |  |  |
| **B11 How many days did you drinking beverages in a typical week during the past 12 months，？**  □ Don’t drink currently → jump to B11a since □ □ year old stop drinking | | | | | | | | | | | | | | | | |  |  |
| □ 1-2 day(s)/week  □ 3-5 days/week  □ Daily/almost everyday | | | | |  | | | | | | |  |  | | | |  |  |
| **B12** | **What kind of drink do you drink most often and what is the frequency？ (choose one)** | | | | | | | | | | | | | | | |  |  |
| Sweeten beverage | | | | | | | | | | | | □ □ cups/week | | | | |  |  |
| Coffee and caffeine beverage | | | | |  | | | | | | | □ □ cups/week | | | | |  |  |
| Others | | | | |  | | | | | | | □ □ cups/week | | | | |  |  |
|  | | | | | | | | | | | | | | | | |  |  |
| **Diets** | | | | | | | | | | | | | | | | |  |  |
| **C1** | |  |  |  | |  | |  |  |  | | | | | |  |  |  |
| **C1a How many people usually eat breakfast together in your family during the past month？** □ □ | | | | | | | | | | | | | | | | |  |  |
| **C1b How many people usually eat lunch together in your family during the past month？ □ □** | | | | | | | | | | | | | | | | |  |  |
| **C1c How many people usually eat dinner together in your family during the past month？** □ □ | | | | | | | | | | | | | | | | |  |  |
| **C2 What kind of oil be used mostly in your family？（choose at most two）** | | | | | | | | | | | | | | | | |  |  |
| □ Rapeseed oil/Sesame oil kg/month | | | | | | | | | | | | | | | | |  |  |
| □ Peanut oil kg/month | | | | | | | | | | | | | | | | |  |  |
| □ Soybean oil kg/month | | | | | | | | | | | | | | | | |  |  |
| □ Lard oil kg/month | | | | | | | | | | | | | | | | |  |  |
| □ Blended oil kg/month | | | | | | | | | | | | | | | | |  |  |
| □ Others kg/month | | | | | | | | | | | | | | | | |  |  |
| **C3 How many grams salt does your family usually consume per month？** □ grams/month | | | | | | | | | | | | | | | | |  |  |
| **During the past 12 months, how often did you consume the following foods and how much the weight per weight (For individual)！** | | | | | | | | | | | | | | | | |  |  |
|  | | a Eat or not | | b Frequency | | | | | | c Weight per time | | | | | | |  |  |
|  | |  | Yes No | b1 times/d | | b2 times/w | | b3 times/m | b4 times/y | Uncooked | | | | | | Cooked |  |  |
| **C4** | | **Rice** | □ □ | □ | | □ | | □ | □ | — | | | | | | □ g |  |  |
| **C5** | | **Wheat products** | □ □ | □ | | □ | | □ | □ | — | | | | | | □ g |  |  |
| **C6** | | **Coarse grain** | □ □ | □ | | □ | | □ | □ | □ g | | | | | | — |  |  |
| **C7** | | **Tubers** | □ □ | □ | | □ | | □ | □ | □ g | | | | | | — |  | |
| **C8** | | **Meat** | □ □ | □ | | □ | | □ | □ | □ g | | | | | | — |  |  |
| **C9** | | **Poultry** | □ □ | □ | | □ | | □ | □ | □ g | | | | | | — |  |  |
| **C10** | | **Fish/sea food** | □ □ | □ | | □ | | □ | □ | □ g | | | | | | — |  |  |
| **C11** | | **Eggs** | □ □ | □ | | □ | | □ | □ | □ g | | | | | | — |  |  |
| **C12** | | **Fresh vegetables** | □ □ | □ | | □ | | □ | □ | □ g | | | | | | — |  |  |
| **C13** | | **Soybean products** | □ □ | □ | | □ | | □ | □ | □ g | | | | | | — |  |  |
| **C14** | | **Preserved vegetables** | □ □ | □ | | □ | | □ | □ | □ g | | | | | | — |  |  |
| **C15** | | **Fresh fruit** | □ □ | □ | | □ | | □ | □ | □ g | | | | | | — |  |  |
| **C16** | | **Dairy products** | □ □ | □ | | □ | | □ | □ | □ g | | | | | | — |  |  |
| **C17 During the past 12 months, have you taken dietary supplements regularly last over a month？** | | | | | | | | | | | | | | | | | |  |
| □ Yes  □ No | | | | | | |  | | | | | | |  |  | | |  |
| **C18 What kind of dietary supplements have you ever taken?** | | | | | | | | | | | | | | | | | |  |
| **C18a Fish oil/cod liver oil** | | | | | | | □ Yes | | | | | | | □ No | | | |  |
| **C18b Vitamin D** | | | | | | | □ Yes | | | | | | | □ No | | | |  |
| **C18c Other vitamins** | | | | | | | □ Yes | | | | | | | □ No | | | |  |
| **C18d Calcium pills** | | | | | | | □ Yes | | | | | | | □ No | | | |  |
| **C18e Others** | | | | | | | □ Yes | | | | | | | □ No | | | |  |

# Supplementary Table 1-3. The scoring criterion for dietary patterns.

## Supplementary Table 1. Scoring criteria for the AMED score in the CMEC study.

| **Component** | **FFQ item ^a^** | **Foods** | **Scoring criteria** |
| --- | --- | --- | --- |
| Vegetables | C12: fresh vegetables | All fresh vegetables except tubers and legumes | Q1=1 point  Q2=2 points  Q3=3 points  Q4=4 points  Q5=5 points |
| Legumes | C13: soybean products | Soybeans, black beans, tofu, soybean milk, dried beans, dried bean curd |  |
| Fruit | C15: fresh fruit | All fresh fruits |  |
| Whole grains | C6: coarse grain | Oats, sorghum, dried corn, highland barely |  |
| Fish | C10: fish/sea food | Fish and all kinds of seafood products |  |
| MUFA: SFA | C2, C4-13, C16 ^b^ | From all kinds of foods and fats |  |
| Red & processed meats | C8: meat | Beef, mutton, pork and their products | Reverse score:  Q1=5 points  Q2=4 points  Q3=3 points  Q4=2 points  Q5=1 point |
| Ethanol | A4, A6 | All alcoholic beverages | moderate alcohol intake criteria ^c^ |

Abbreviation: AMED for alternative Mediterranean diet; MUFA: SFA for the ratio of monounsaturated fatty acids: saturated fatty acids.

a. See more details in text 1s.

b. Due to there is no values of fatty acids for food groups in the China food exchange list, we made an exchange value table according to the common consumed food items in each food group in Southwest China and the 2018 China food composition tables. See more details in the following table 3s.

c. According to the encouragement of moderate alcohol intake, the alcohol consumptions were categorized into five groups: (10,30], (0,10] or (30,40], 0 or (40,45], (45,50], and >50 grams per day for men; (5,15], (0,5] or (15,25], 0 or (25,30], (30,35], and >35 grams per day for women, and then we assigned descending scores of 1-5 to corresponding individuals.

## Supplementary Table 2. Scoring criteria for the DASH score in the CMEC study.

| **Component** | **FFQ item ^a^** | **Foods** | **Scoring criteria** |
| --- | --- | --- | --- |
| Fruit | C15: fresh fruit | All fresh fruit | Q1=1 point  Q2=2 points  Q3=3 points  Q4=4 points  Q5=5 points |
| Vegetable | C12: fresh vegetables | All fresh vegetables except tubers and legumes |  |
| Legumes | C13: soybean products | Soybeans, black beans, tofu, soybean milk, dried beans, dried bean curd |  |
| Dairy Product | C16: dairy products | Fresh milk, yogurt, cheese, milk tea |  |
| Whole Grains | C6: coarse grain | Oats, sorghum, dried corn, highland barely |  |
| Red & processed meat | C8: meat | Beef, mutton, pork and their products | Reverse score:  Q1=5 points  Q2=4 points  Q3=3 points  Q4=2 points  Q5=1 point |
| Sodium | C1, C3, C14 | Sodium in salt and preserved vegetables |  |

Abbreviation: DASH for Dietary Approaches to Stop Hypertension.

a. See more details in text 1s.

## Supplementary Table 3. Exchange values of fatty acids for food groups included in the CMEC study ^a^

| **Food group** | **Unit (g)** | **SFA (g)** | **MUFA (g)** | **PUFA (g)** | **MUFA: SFA** |
| --- | --- | --- | --- | --- | --- |
| Rice | 300 | 0.3 | 0.2 | 0.3 | 0.7 |
| Wheat products | 100 | 0.4 | 0.2 | 0.1 | 0.4 |
| Coarse grain | 100 | 0.8 | 1.0 | 0.5 | 1.3 |
| Tubers | 100 | 0.0 | 0.1 | 0.1 | - |
| Pork ^b^ | 100 | 10.8 | 13.3 | 2.1 | 1.2 |
| Beef ^c^ | 100 | 4.1 | 3.5 | 0.3 | 0.9 |
| Mutton ^d^ | 100 | 4.2 | 2.4 | 0.8 | 0.6 |
| Poultry | 100 | 4.7 | 7.7 | 3.0 | 1.6 |
| Fish/Sea food | 100 | 0.7 | 0.9 | 0.5 | 1.3 |
| Dairy products | 100 | 2.1 | 1.0 | 0.1 | 0.5 |
| Eggs | 100 | 4.6 | 1.9 | 0.5 | 0.4 |
| Soybean products | 100 | 2.4 | 3.5 | 9.1 | 1.5 |
| Vegetable oil | 100 | 13.4 | 40.0 | 40.2 | 3.0 |
| Animal oil | 100 | 41.1 | 45.6 | 8.5 | 1.1 |

Abbreviation: SFA: saturated fatty acids; MUFA: monounsaturated fatty acids; PUFA: polyunsaturated fatty acids; MUFA: SFA for the ratio of monounsaturated fatty acids: saturated fatty acids.

a. The saturated and unsaturated fatty acid content in unit of each food group was calculated by averaging that of common food items in the southwest China in the same group by referring to 2018 China.

b-d. We considered the consumed proportion of different types of red meats when calculating the fatty acids of meats in different regions, due to the major type of red meat consumed differ in different regions.

# Supplementary Methods: directed acyclic graphs (DAG)

According to the protocol of “Evidence Synthesis for Constructing Directed Acyclic Graphs” (ESC-DAGs)^1^, we constructed a directed acyclic graph (DAG) representing the existing literature to select a minimally sufficient set of confounders. Firstly, based on systematic literature review, we initially identified all potential confounders between dietary pattern and metabolic dysfunction-associated fatty liver disease (MAFLD). Secondly, we created a saturated ‘implied graph’ (IG) by drawing directed or undirected edges between all confounders. The IG acted as a transparent structural template for translation into a DAG. Thirdly, we assessed whether each directed edge in the IG is feasible for three causal criteria (i.e., temporality, face-validity and recourse to theory), thus translating the IG into a DAG. At the same time as the DAG was constructed, a series of conditional independences were generated. Fourthly, we continuously conducted the independence test with our data and modified the DAG at the same time, until all the implied conditional independences were satisfied. Lastly, the final DAG was constructed and a minimally sufficient set of confounders was selected. A software program called DAGitty was used to draw and interpret DAG (http://www. dagitty.net/dags.html)^2^.

Based on the DAG, the following variables were retained as confounders in our statistical models: age (years), sex (male or female), marital status (married/cohabiting or not), highest education attained (no formal school, primary school, middle and high school, college/university or higher), household income (<¥12000, ¥12000-19999, ¥20000-5999, ¥60000-99999, ¥100000-199999, > ¥200000), profession (primary industry practitioner, secondary industry practitioner, tertiary industry practitioner, unemployed), regular smoking (never, former, current), physical activity in metabolic equivalent tasks (METs), total energy intake (kcal per day), regular intake of sweeten beverage (never, former, current), insomnia symptoms (yes or no), depressive symptom (yes or no), anxiety symptom (yes or no), menopause status for women (premenopausal, perimenopausal, postmenopausal), and family history of cardiometabolic diseases (yes or no), BMI (<25 kg/m^2^ or ≥25 kg/m^2^). Additionally, we adjusted for regional level confounders, i.e., urbanicity (urban or rural) and ethnicity (Han, Tibetan, Yi, Miao, Bai, Bouyei, Dong), and dieted-related variables, i.e., regular intake of dietary supplements (yes or no), regular intake of spicy food (yes or no), and regular intake of pepper food (yes or no).


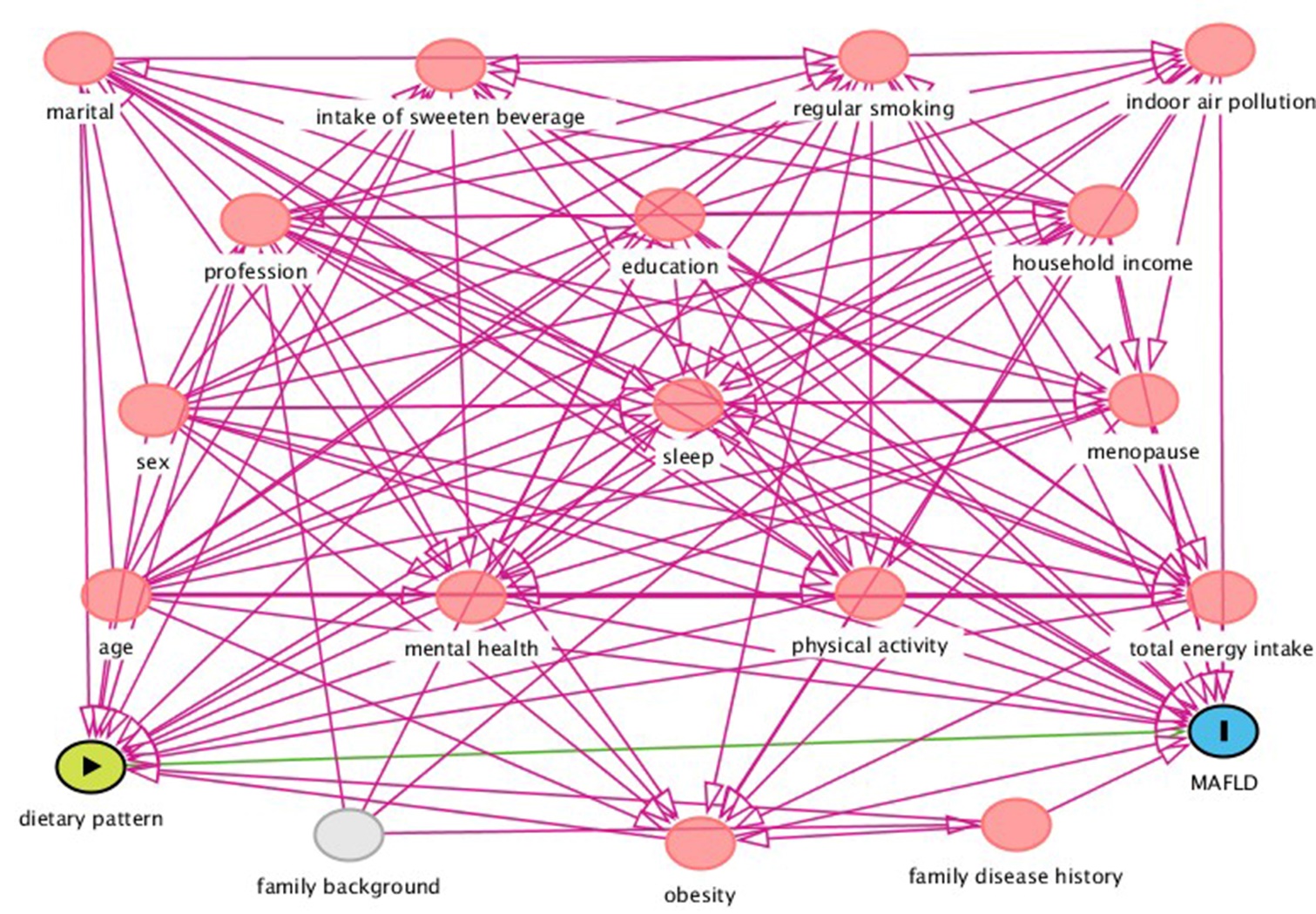


# Supplementary Figure 1: The final constructed DAG.

# Supplementary Table 4-8: Sensitivity analyses

## Supplementary Table 4: Estimated associations using 24 kg/m^2^ and 28 kg/m^2^ as the BMI cutoff value of lean or overweight or obese individuals (n = 66,526).

|  | **ALL MAFLD** | | |  | | **Nonobese MAFLD** | |  | **Obese MAFLD** | | **Heterogeneity Test ^a^** |
| --- | --- | --- | --- | --- | --- | --- | --- | --- | --- | --- | --- |
|  | **No. of Case** | **OR (95% CI)** |  | | **No. of Case** | | **OR (95% CI)** |  | **No. of Case** | **OR (95% CI)** |  |
| **AMED** |  |  | |  | |  |  |  |  |  |  |
| Quintile 1 | 1836 | 1 (Reference) | |  | | 1026 | 1 (Reference) |  | 582 | 1 (Reference) | I^2^= 1.0%; P=0.886 |
| Quintile 2 | 2250 | 1.00(0.94,1.07) | |  | | 1414 | 1.01(0.93,1.10) |  | 778 | 0.99(0.86,1.15) |  |
| Quintile 3 | 1773 | 0.97(0.90,1.03) | |  | | 1192 | 1.01(0.93,1.09) |  | 594 | 1.00(0.86,1.16) |  |
| Quintile 4 | 1761 | 1.00(0.94,1.07) | |  | | 1220 | 1.07(0.99,1.16) |  | 814 | 0.99(0.85,1.15) |  |
| Quintile 5 | 2832 | 0.97(0.91,1.04) | |  | | 1921 | 1.03(0.95,1.12) |  | 911 | 0.98(0.84,1.13) |  |
| *P* trend |  | 0.322 | |  | |  | 0.291 |  |  | 0.758 |  |
| **DASH** |  |  | |  | |  |  |  |  |  |  |
| Quintile 1 | 1966 | 1 (Reference) | |  | | 1236 | 1 (Reference) |  | 536 | 1 (Reference) | I^2^= 63.6%; P=0.027 |
| Quintile 2 | 1527 | 0.98(0.92,1.05) | |  | | 1507 | 0.92(0.85,0.99) |  | 770 | 0.96(0.83,1.11) |  |
| Quintile 3 | 2763 | 0.96(0.90,1.02) | |  | | 1183 | 0.87(0.80,0.94) |  | 695 | 1.09(0.94,1.26) |  |
| Quintile 4 | 1739 | 0.94(0.88,1.00) | |  | | 1614 | 0.85(0.79,0.92) |  | 897 | 0.96(0.83,1.12) |  |
| Quintile 5 | 2457 | 0.86(0.80,0.92) | |  | | 1233 | 0.76(0.70,0.82) |  | 781 | 0.94(0.81,1.09) |  |
| *P* trend |  | < 0.001 | |  | |  | < 0.001 |  |  | 0.368 |  |

MAFLD, metabolic dysfunction-associated fatty liver disease; AMED, Alternate Mediterranean diet; DASH, Dietary Approaches to Stop Hypertension; OR, odds ratio; CI, confidence interval.

^a^ Heterogeneity tests were performed between nonobese MAFLD and obese MAFLD.

## Supplementary Table 5: Estimated associations in participants without exclusion of self-reported physician diagnosed chronic hepatitis/cirrhosis, diabetes, hypertension, hyperlipidemia, coronary heart disease, stroke or cancer (n = 91,452).

|  | **ALL MAFLD** | | |  | | **Nonobese MAFLD** | |  | **Obese MAFLD** | | **Heterogeneity Test ^a^** |
| --- | --- | --- | --- | --- | --- | --- | --- | --- | --- | --- | --- |
|  | **No. of Case** | **OR (95% CI)** |  | | **No. of Case** | | **OR (95% CI)** |  | **No. of Case** | **OR (95% CI)** |  |
| **AMED** |  |  | |  | |  |  |  |  |  |  |
| Quintile 1 | 3189 | 1 (Reference) | |  | | 625 | 1 (Reference) |  | 2564 | 1 (Reference) | I^2^= 14.3%;  P= 0.323 |
| Quintile 2 | 2506 | 1.00(0.95,1.05) | |  | | 880 | 1.00(0.90,1.10) |  | 1952 | 0.99(0.93,1.06) |  |
| Quintile 3 | 4675 | 1.00(0.95,1.05) | |  | | 787 | 1.06(0.96,1.17) |  | 3562 | 0.99(0.93,1.06) |  |
| Quintile 4 | 3086 | 1.01(0.96,1.06) | |  | | 758 | 1.02(0.93,1.13) |  | 2328 | 1.00(0.94,1.07) |  |
| Quintile 5 | 4927 | 0.96(0.91,1.01) | |  | | 1258 | 1.02(0.93,1.13) |  | 3669 | 0.92(0.86,0.98) |  |
| *P* trend |  | 0.126 | |  | |  | 0.478 |  |  | 0.022 |  |
| **DASH** |  |  | |  | |  |  |  |  |  |  |
| Quintile 1 | 3519 | 1 (Reference) | |  | | 787 | 1 (Reference) |  | 1953 | 1 (Reference) | I^2^= 79.7%; P=0.001 |
| Quintile 2 | 2674 | 0.97(0.92,1.02) | |  | | 905 | 0.86(0.79,0.95) |  | 2909 | 0.99(0.93,1.06) |  |
| Quintile 3 | 4827 | 0.97(0.92,1.02) | |  | | 759 | 0.86(0.78,0.95) |  | 2482 | 1.01(0.95,1.08) |  |
| Quintile 4 | 2922 | 0.90(0.85,0.95) | |  | | 1019 | 0.80(0.73,0.88) |  | 3414 | 0.91(0.85,0.97) |  |
| Quintile 5 | 4441 | 0.87(0.83,0.92) | |  | | 838 | 0.69(0.62,0.76) |  | 3317 | 0.90(0.84,0.96) |  |
| *P* trend |  | < 0.001 | |  | |  | < 0.001 |  |  | < 0.001 |  |

MAFLD, metabolic dysfunction-associated fatty liver disease; AMED, Alternate Mediterranean diet; DASH, Dietary Approaches to Stop Hypertension; OR, odds ratio; CI, confidence interval.

^a^ Heterogeneity tests were performed between nonobese MAFLD and obese MAFLD.

## Supplementary Table 6: Estimated associations using logistic regression (n=66,526).

|  | **Overall MAFLD** | |  | **Nonobese MAFLD** | |  | **Obese MAFLD** | | **Heterogeneity Test ^a^** |
| --- | --- | --- | --- | --- | --- | --- | --- | --- | --- |
|  | **No. of Case** | **OR (95% CI)** |  | **No. of Case** | **OR (95% CI)** |  | **No. of Case** | **OR (95% CI)** |  |
| **AMED** |  |  |  |  |  |  |  |  |  |
| Quintile 1 | 1,863 | 1 (Reference) |  | 377 | 1 (Reference) |  | 1,486 | 1 (Reference) | *I^2^*=1.0%; *P*=0.424 |
| Quintile 2 | 2,301 | 1.03 (0.95-1.11) |  | 549 | 1.04 (0.90-1.19) |  | 1,752 | 1.03 (0.94-1.13) |  |
| Quintile 3 | 1,824 | 1.00 (0.92-1.08) |  | 494 | 1.09 (0.95-1.26) |  | 1,330 | 0.95 (0.86-1.05) |  |
| Quintile 4 | 1,802 | 1.02 (0.94-1.11) |  | 491 | 1.09 (0.95-1.27) |  | 1,311 | 1.00 (0.90-1.10) |  |
| Quintile 5 | 2,905 | 0.99 (0.91-1.07) |  | 795 | 1.05 (0.91-1.21) |  | 2,110 | 0.96 (0.87-1.06) |  |
| *P* trend |  | 0.158 |  |  | 0.502 |  |  | 0.260 |  |
| **DASH** |  |  |  |  |  |  |  |  |  |
| Quintile 1 | 2,011 | 1 (Reference) |  | 493 | 1 (Reference) |  | 1,518 | 1 (Reference) | *I^2^*=58.5%; *P*=0.047 |
| Quintile 2 | 1,551 | 0.96 (0.89-1.05) |  | 341 | 0.82 (0.71-0.95) |  | 1,210 | 1.05 (0.95-1.15) |  |
| Quintile 3 | 2,821 | 0.95 (0.88-1.02) |  | 700 | 0.85 (0.75-0.96) |  | 2,121 | 1.00 (0.92-1.09) |  |
| Quintile 4 | 1,780 | 0.92 (0.85-1.00) |  | 478 | 0.85 (0.74-0.97) |  | 1,302 | 0.96 (0.87-1.06) |  |
| Quintile 5 | 2,532 | 0.79 (0.73-0.85) |  | 694 | 0.68 (0.60-0.78) |  | 1,838 | 0.85 (0.77-0.93) |  |
| *P* trend |  | < 0.001 |  |  | < 0.001 |  |  | <0.001 |  |

MAFLD, metabolic dysfunction-associated fatty liver disease; AMED, Alternate Mediterranean diet; DASH, Dietary Approaches to Stop Hypertension; OR, odds ratio; CI, confidence interval.

^a^ Heterogeneity tests were performed between nonobese MAFLD and obese MAFLD.

## Supplementary Table 7: Estimated associations with the complete case instead of imputed data (n=48,989).

|  | **ALL MAFLD** | | |  | | **Nonobese MAFLD** | |  | **Obese MAFLD** | | **Heterogeneity Test ^a^** |
| --- | --- | --- | --- | --- | --- | --- | --- | --- | --- | --- | --- |
|  | **No. of Case** | **OR (95% CI)** |  | | **No. of Case** | | **OR (95% CI)** |  | **No. of Case** | **OR (95% CI)** |  |
| **AMED** |  |  | |  | |  |  |  |  |  |  |
| Quintile 1 | 1,381 | 1 (Reference) | |  | | 266 | 1 (Reference) |  | 1115 | 1 (Reference) | I^2^= 61.9%; P=0.033 |
| Quintile 2 | 1,062 | 1.01(0.94,1.09) | |  | | 399 | 1.05(0.91,1.21) |  | 804 | 0.97(0.88,1.07) |  |
| Quintile 3 | 1,980 | 0.99(0.91,1.06) | |  | | 375 | 1.13(0.98,1.31) |  | 1464 | 0.92(0.83,1.02) |  |
| Quintile 4 | 1,287 | 0.99(0.92,1.07) | |  | | 353 | 1.11(0.96,1.28) |  | 934 | 0.92(0.83,1.01) |  |
| Quintile 5 | 2,098 | 0.94(0.87,1.01) | |  | | 591 | 1.08(0.94,1.25) |  | 1507 | 0.84(0.76,0.93) |  |
| *P* trend |  | 0.077 | |  | |  | 0.194 |  |  | < 0.001 |  |
| **DASH** |  |  | |  | |  |  |  |  |  |  |
| Quintile 1 | 1,476 | 1 (Reference) | |  | | 351 | 1 (Reference) |  | 805 | 1 (Reference) | I^2^= 56.2%; P=0.058 |
| Quintile 2 | 1,156 | 1.00(0.93,1.08) | |  | | 255 | 0.84(0.74,0.97) |  | 1221 | 1.03(0.93,1.14) |  |
| Quintile 3 | 2,073 | 0.97(0.90,1.05) | |  | | 539 | 0.92(0.80,1.05) |  | 1032 | 1.00(0.90,1.10) |  |
| Quintile 4 | 1,284 | 0.94(0.88,1.02) | |  | | 462 | 0.78(0.68,0.90) |  | 1459 | 0.95(0.85,1.05) |  |
| Quintile 5 | 1,819 | 0.88(0.82,0.95) | |  | | 377 | 0.72(0.62,0.83) |  | 1307 | 0.89(0.81,0.99) |  |
| *P* trend |  | < 0.001 | |  | |  | < 0.001 |  |  | 0.008 |  |

MAFLD, metabolic dysfunction-associated fatty liver disease; AMED, Alternate Mediterranean diet; DASH, Dietary Approaches to Stop Hypertension; OR, odds ratio; CI, confidence interval.

^a^ Heterogeneity tests were performed between nonobese MAFLD and obese MAFLD.

## Supplementary Table 8: Adjusted associations between AMED and DASH and NAFLD (n = 66,526).

|  | **ALL NAFLD** | | |  | | **Nonobese NAFLD** | |  | **Obese NAFLD** | | **Heterogeneity Test ^a^** |
| --- | --- | --- | --- | --- | --- | --- | --- | --- | --- | --- | --- |
|  | **No. of Case** | **OR (95% CI)** |  | | **No. of Case** | | **OR (95% CI)** |  | **No. of Case** | **OR (95% CI)** |  |
| **AMED** |  |  | |  | |  |  |  |  |  |  |
| Quintile 1 | 1756 | 1 (Reference) | |  | | 361 | 1 (Reference) |  | 1395 | 1 (Reference) | I^2^ = 60.5%;  P = 0.038 |
| Quintile 2 | 2166 | 1.00(0.94,1.07) | |  | | 550 | 0.99(0.88,1.13) |  | 1027 | 1.02(0.93,1.11) |  |
| Quintile 3 | 1757 | 1.01(0.95,1.08) | |  | | 513 | 1.10(0.97,1.24) |  | 1833 | 0.97(0.89,1.06) |  |
| Quintile 4 | 1715 | 1.02(0.96,1.09) | |  | | 500 | 1.06(0.94,1.20) |  | 1215 | 1.00(0.92,1.10) |  |
| Quintile 5 | 2805 | 1.01(0.94,1.08) | |  | | 852 | 1.12(1.00,1.27) |  | 1953 | 0.94(0.86,1.03) |  |
| *P* trend |  | 0.731 | |  | |  | 0.025 |  |  | 0.133 |  |
| **DASH** |  |  | |  | |  |  |  |  |  |  |
| Quintile 1 | 1843 | 1 (Reference) | |  | | 462 | 1 (Reference) |  | 1381 | 1 (Reference) | I^2^ = 73.5%;  P = 0.005 |
| Quintile 2 | 1457 | 0.98(0.92,1.05) | |  | | 571 | 0.84(0.75,0.94) |  | 1117 | 1.08(0.99,1.17) |  |
| Quintile 3 | 2696 | 0.97(0.91,1.03) | |  | | 500 | 0.86(0.77,0.96) |  | 1315 | 1.04(0.95,1.13) |  |
| Quintile 4 | 1709 | 0.93(0.87,1.00) | |  | | 667 | 0.79(0.70,0.88) |  | 1869 | 0.97(0.89,1.06) |  |
| Quintile 5 | 2494 | 0.86(0.80,0.92) | |  | | 576 | 0.72(0.63,0.81) |  | 1741 | 0.91(0.83,0.99) |  |
| *P* trend |  | < 0.001 | |  | |  | < 0.001 |  |  | 0.003 |  |

NAFLD, nonalcoholic fatty liver disease; AMED, Alternate Mediterranean diet; DASH, Dietary Approaches to Stop Hypertension; OR, odds ratio; CI, confidence interval.

^a^ Heterogeneity tests were performed between nonobese NAFLD and obese NAFLD.

**References**

1. Ferguson KD, McCann M, Katikireddi SV, et al. Evidence synthesis for constructing directed acyclic graphs (ESC-DAGs): a novel and systematic method for building directed acyclic graphs. *Int J Epidemiol* 2020;49(1):322-29. doi: 10.1093/ije/dyz150 [published Online First: 2019/07/22]

2. Textor J, Hardt J, Knüppel S. DAGitty: a graphical tool for analyzing causal diagrams. *Epidemiology (Cambridge, Mass)* 2011;22(5):745. doi: 10.1097/EDE.0b013e318225c2be
